# Toxic Effects of Koumine on the Early-Life Development Stage of Zebrafish

**DOI:** 10.3390/toxics11100853

**Published:** 2023-10-12

**Authors:** Dongjie Wang, Xinyi Leng, Yao Tian, Jiangdong Liu, Jixing Zou, Shaolin Xie

**Affiliations:** 1 College of Marine Sciences, South China Agricultural University, Guangzhou 510642, China; dongjiewang1995@gmail.com; 2 College of Life Sciences, Wuhan University, Wuhan 430000, China; uk19940329@163.com (X.L.); liujd@whu.edu.cn (J.L.); 3 Global Health Institute, School of Life Science, École Polytechnique Fédérale de Lausanne (EPFL), 1015 Lausanne, Switzerland; ty19981214@gmail.com

**Keywords:** koumine, developmental toxicity, behavioral neurotoxicity, AChE

## Abstract

Koumine is one of the most abundant alkaloids found in *Gelsemium elegans*, and it has a wide range of pharmacological effects including antitumor, anti-inflammatory, analgesic treatment effects, and antianxiety. However, its high toxicity and unclear mechanism of action have greatly limited the medicinal development and use of koumine. We investigated the toxic effects of koumine on the developmental toxicity and behavioral neurotoxicity of zebrafish embryos and larvae. Embryos at 6 h postfertilization (hpf) were exposed to 12.5, 25, 50, 75, and 100 mg/L of koumine until 120 hpf. Koumine affected the hatching and heartbeats of the embryos. The morphological analysis also revealed many abnormalities, such as shortened bodies, yolk sac edemas, tail malformations, and pericardial edemas. To identify the neurotoxicity of koumine, the behavior of the larvae was measured. Koumine at 50 and 100 mg/L affect the escape response. The embryos exhibited uncoordinated muscle contractions along the body axis in response to touch at 36 hpf. More importantly, we found that the neurotoxicity of koumine is mainly caused by influencing the ACh content and the activity of AChE without impairing motor neuron development. A comprehensive analysis shows that a high concentration of koumine has obvious toxic effects on zebrafish, and the safe concentration of koumine for zebrafish should be less than 25 mg/L. These results will be valuable for better understanding the toxicity of koumine and provide new insights into the application of koumine.

## 1. Introduction

*Gelsemium elegans*, known as heartbreak grass, is widely distributed in China and Southeast Asian countries. Phytochemical studies have revealed that *G. elegans* is rich in alkaloids, especially indole alkaloids [1]. Four alkaloids have been extracted mainly from *G. elegans*, including gelsemine, koumine, gelsevirine, and gelsenicine [2]. Koumine is one of the most abundant alkaloids found in *G. elegans* and has a wide range of pharmacological effects [3,4]. In addition to its antitumor, anti-inflammatory, and analgesic treatment effects, koumine is also an effective antianxiety agent [1]. Studies have shown that koumine, administered subcutaneously to mice, has a significant anxiolytic effect in the light–dark transition test (LDT) and the open-field test (OFT). Koumine has also shown significant anxiolytic effects in mice in the functional observation battery (FOB) and Vogel conflict test (VCT) but did not produce any adverse effects on the nervous system [5]. It has been suggested that the anxiolytic effect of koumine may be related to neurosteroid substances, with koumine promoting the synthesis of tetrahydroprogesterone and progesterone in the brain and reducing the levels of adrenocorticotropic hormones and corticosteroids [6].

Koumine also has toxic effects [7]. Studies have shown that *G. elegans* alkaloids have a very pronounced toxic effect on the central nervous system, with animals suffering from reduced locomotor activity, a reduced respiratory rate, violent tremors and clonic convulsions, and even death by asphyxiation in respiratory arrest [8]. The pharmacokinetics of koumine showed that koumine was distributed in all tissues of mouse (*Mus musculus*) after gavage and had severe toxic effects on the liver, kidneys, and testes. Koumine can cross the blood–brain barrier and was eliminated relatively slowly in the brain, suggesting that koumine may have a strong neurotoxic effect in the nervous system [9]. However, there is little information about the toxic effects of koumine, which greatly limits the application of it.

The zebrafish (*Danio rerio*) is a vertebrate animal model that is increasingly being used for in vivo drug toxicity and efficacy screening and for the assessment of chemical toxicity [10]. The US Environmental Protection Agency (EPA) has developed guidelines for the detection of developmental toxicity assays in zebrafish that specify toxic endpoints during zebrafish development, including abnormalities in the hatching rate, mortality, heart rate, morphology, and behavior, providing uniform criteria for assessing the developmental toxicity of compounds [11]. The multiple strengths of zebrafish also make them well suited for neurobehavioral studies. For example, zebrafish have a set behavioral patterns during early development, with spontaneous tail curling, touch avoidance responses, and spontaneous swimming behavior occurring in sequence; in addition, zebrafish embryos develop rapidly, with the sensory and motor systems of larvae maturing by 5 dpf, when they can display a variety of complex neurobehaviors such as tropism and startle avoidance reflexes in response to bright light stimuli [12].

Studies have been conducted to confirm the potential neurological effects of koumine, but the underlying mechanisms are unclear. Acetylcholinesterase (AChE) is primarily responsible for the hydrolysis of the synaptic gap neurotransmitter ACh in the cholinergic system, which is an enzyme essential for the proper functioning of the nervous system, especially the cholinergic system [13]. Taken together, we speculate that ACh may be one of the regulatory targets of koumine. In this study, we investigated the developmental toxicity and behavioral neurotoxicity induced by koumine in zebrafish embryos. More importantly, our experimental results revealed that the neurotoxicity of koumine is mainly caused by influencing the ACh content and the activity of AChE without impairing motor neuron development. The results obtained from this study will be valuable for better understanding the toxicity of koumine and provide new insights into the application of koumine.

## 2. Materials and Methods

### 2.1. Zebrafish Husbandry

Wild-type zebrafish (AB strain, 3 months old) were obtained from the Institute of Hydrobiology, Chinese Academy of Science (Wuhan, Hubei, China), and were maintained according to standard husbandry procedures (Westerfield 2007) at 28 °C. Adult zebrafish were kept in a water circulation system with 14 h of light and 10 h of darkness (pH = 7.1 ± 0.2, DO = 6.7 ± 0.5 mg/L, EC = 278 ± 37). The zebrafish were fed with hatching *Artemia salina* every morning and evening. The night before spawning, sexually mature male and female zebrafish were placed in the spawning box with a partition separating the male and female zebrafish, and the next morning, the partition was removed, and the zebrafish began to chase their tails and spawn under the stimulation of light. Embryonic stages were represented by using the unit hour postfertilization (hpf). The embryos were collected during natural spawning periods and were cultured in Hank’s solution prior to further experimentation [14]. The embryos were examined under a stereomicroscope (SMZ45, Sunny Optical, Ningbo, China).

### 2.2. Chemicals

Koumine (98% purity,1358-76-5) was obtained from the Must Biological Company (Chengdu, China) and dissolved in Hank’s solution to prepare stock solutions of 500 mg/L. Dilutions were prepared daily from stock solutions. The stock solutions were sampled for analyses of koumine during the assay at 0, 24, 48, 72, 96, and 120 h. All the analyses were performed by using high-performance liquid chromatography (Agilent ZORBAX SB-C18). The HPLC column used is a C18 column, 15 cm in length and 0.46 cm in internal diameter. The mobile phase was acetonitrile: 0.1% H_3_PO_4_ (the pH was adjusted with triethylamine to 7–8) and water (40:40:20). The limit of detection (LOD) was estimated at 14 mg/L (koumine). A quantitative analysis was conducted on a five-point linear calibration of the koumine solutions. The koumine concentration assay was performed only for the stock solutions, which were subsequently diluted to working concentrations.

### 2.3. Exposures

To determine the effects of koumine on the functional, behavioral, and morphological aspects of zebrafish development, fertilized and normal embryos (6 hpf) were randomly chosen and transferred into 6-well plates (15 embryos per well) containing 2 mL solutions with different concentrations of koumine (12.5, 25, 50, 75, and 100 mg/L). The concentration selection and rationality were carried out with reference to similar studies [15,16]. All the experiments were repeated three times with a control group independently. During 120 hpf exposure, the zebrafish larvae were screened for morphological abnormalities under a stereomicroscope, and the hatching rate, heart rate, and body length were recorded for each treatment group. Their heart rate was recorded at 72 hpf for the control and koumine-group zebrafish larvae, and their body length was measured at 120 hpf. Ten larvae fish were randomly selected from each group for the body size and heart rate measurements. The exposures were static and the solutions were renewed every 24 h. During the exposure, abnormal and dead embryos were removed from the plates. Blank control groups (Hank’s solution) were set at the same time. The exposure and experimental procedures are illustrated in Figure S1.

### 2.4. Development Toxicity Analyses

During the 120 hpf exposure, the embryos were examined under a stereomicroscope to screen for morphological abnormalities, and hatching rates were recorded within each treatment. Similarly, additional experiments were performed in which the embryos were subjected to heart rate and length measurements and behavioral detection after treatment. Ten larvae randomly selected from each treatment concentration and blank control group were analyzed with a stereomicroscope to measure the total larva length. The length of each embryo along the body axis from the anterior-most part of the head to the tip of the tail was measured by using digital images produced with ImageJ (Media Cybernetics, Bethesda, MD, USA). The heart rates of ten zebrafish larvae were counted at 72 hpf by visual observation at 20 s intervals under a stereomicroscope at an appropriate ambient temperature (T = 26 ± 1 °C). The embryos were examined daily for hatching. When the head or tail of a larva broke out of the embryonic membrane, hatching was considered to be successful. Heart rate statistics and body length measurement experiments were performed in three replications.

### 2.5. Behavioral Analyses

Two early behaviors were chosen for the determination of behavioral toxicity. The spontaneous behavior of dechorionated embryos was observed at 20 hpf (*n* = 10). Hatched larvae were gently touched on the tail with forceps at 27 and 36 hpf. The response to a mechanical stimulus (touch) was used as a measure of sensorimotor integration [17].

To determine the effect of low concentrations of koumine on the swimming behavior of zebrafish larvae, we conducted independent exposure experiments. Embryos (6 hpf) were exposed to 20 mg/L of koumine for up to 120 hpf. This concentration on the larvae fish did not cause significant deformities. For these exposures, no obvious malformations were observed. The quantification of locomotor activity was achieved by using VideoTrack for Zebrafish TM (version 3.5, with background subtraction; Viewpoint, France). At 96 hpf, 10 individual larvae in each concentration were individually transferred into 24-well plates (1 larva per well) containing 2 mL of Hank’s solution. Behavioral testing began on 5 dpf at 12:00 when the activity was relatively stable. Free swimming activities were detected and recorded in continuous darkness (3 min). The experiments were performed in a calm, sealed area. The larvae were allowed to adjust to the dark environment for approximately 10 min before recording was started. The swimming pattern of each larva was recorded every minute, and the total observation time was 3 min. The total distance was calculated. This value characterizes the general swimming ability of the larvae. The control group was untreated larvae. The behavioral experiments were repeated three times.

### 2.6. Muscle Morphology

Zebrafish larvae were collected at 120 hpf at an exposure concentration of 100 mg/L of koumine and fixed in 4% paraformaldehyde for 24 h. The samples were dehydrated in alcohol and xylene and then embedded in paraffin. The embedded samples were sliced on a microtome to a thickness of 4 μm. Hematoxylin and eosin stains were used for staining. Histological images were photographed by an Automatic Digital Slide Scanning System (M8, Precipoint, München, Germany).

### 2.7. Neurotransmitter Content

The zebrafish larvae exposed to 50 mg/L of koumine at 96 hpf were collected for the determination of acetylcholine by high-performance liquid chromatography–tandem mass spectrometry. Each group was sampled at 150 mg and replicated three times. The ACh standards were prepared in 80% methanol as a high-concentration working solution and subsequently diluted and assayed stepwise, and the standard curve was finally calculated. Chromatographic conditions: ACQUITY UPLC^®^BEH C18 column (2.1 × 100 mm, 1.7 μm, Waters Corporation, Milford, MA, USA), injection volume 5 μL, column temperature 40 °C, and mobile phase A was 10% methanolic water (containing 0.1% formic acid) and B was 50% methanolic water (containing 0.1% formic acid). The gradient elution conditions were 0~6.5 min, 10~30% B; 6.5~7 min, 30~100% B; 7~12 min, 100% B; 12~12.5 min, 100~10% B; and 12.5~16.5 min, 10% B. The flow rates were 0~12.5 min, 0.3 mL/min and 12.5~16.5 min, 0.3~0.4 mL/min. Mass spectrometry conditions: electrospray ionization (ESI) source, positive ionization mode. The ion source temperature was 500 °C, ion source voltage was 5000 V, collision gas was 6 psi, curtain gas was 30 psi, and atomization gas and auxiliary gas was 50 psi. Scans were performed by using multiple reaction monitoring (MRM). DP: 51, EP: 10, CE: 19, CXP: 6. The concentration of the standard was used as the horizontal coordinate, and the peak area was used as the vertical coordinate. The linear regression equation of ACh was obtained as Y = 17,700X + 2830. R = 0.9949, linearity range 0.25–500 ng/mL, quantization limit 0.25 ng/mL.

### 2.8. AChE Activity Assay

Due to the toxic effects of koumine, the sample size of the high concentration group (75, 100 mg/L) at the later stages of exposure could no longer meet the sampling requirements. We selected the remaining group for follow-up experiments (100 mg). The distribution of the total protein and ACh measurements was performed by using the BCA protein concentration assay kit and the acetylcholinesterase (AChE) assay kit (Nanjing Jiancheng Bioengineering Institute, Nanjing, China). Specifically, 100 mg (*n* = 3) of larvae fish from each of the 96 hpf toxicity groups and the control group were collected, added to 0.1 mL of saline, and homogenized manually on ice. The homogenate was centrifuged at 2500 rpm for 10 min and the supernatant was extracted and tested according to the kit instructions. The determination of the total protein content was performed according to the instructions, and a portion of the supernatant was diluted 10 fold. AChE and total protein ODs were measured at 405 nm and 562 nm, respectively.

Total protein concentration (μg/mL) = (measured OD value − blank OD value) ÷ (standard OD value − blank OD value) × standard concentration (563 μg/mL) × presample dilution concentration

AChE (U/mg prot) = (measured OD value − control OD value) ÷ (standard OD value − blank OD value) × standard concentration (1 μmol/mL) ÷ total protein concentration of the sample to be tested

Activity unit definition: one enzyme activity unit is defined as the amount of enzyme that catalyzes the production of 1 nmol TNB per minute per milligram of protein.

### 2.9. Observation of Motor Neuron Axon

The 6 hpf embryos were exposed to 100 mg/L of koumine. At 20 hpf, 0.003% PTU was added to the solution to prevent pigmentation. Subsequently, the chorion was removed, and the collected embryos were fixed in 4% paraformaldehyde at 4 °C for 12–24 h. Then, the collected embryos were washed three times with PBST and permeabilized with acetone for 10 min at 4 °C. The embryos were blocked for 1 h with 10% goat serum (diluted with PBST); anti-SYT2 (Znp-1) (1:2000) was added (diluted with 10% goat serum) overnight at 4 °C. The embryos were washed three times with PBST, and we added the secondary antibody (HRP-conjugated antimouse IgG, 200-fold dilution) for 1 h. The embryos were washed three times with PBST, we developed the color by using the DAB kit, and we operated in a dark room. Finally, the growth and development of primary motor neuron axonal caps were observed under a microscope.

### 2.10. Data Analysis

All statistical analyses were performed by using SPSS software (version 17.0, SPSS Inc., Chicago, IL, USA). The data plotting was performed by using GraphPad Prism 6 software (version 6.0, GraphPad Software, San Diego, CA, USA). Prior to a one-way analysis of variance (ANOVA), the homogeneity of variances was tested. Differences in the data were evaluated by a one-way ANOVA with a post hoc least significant difference (LSD) comparison of means. A value of *p* < 0.05 was considered significant. All of the data are recorded as the means ± SD.

## 3. Results

### 3.1. Developmental Toxicity of Koumine

**Figure 1:**
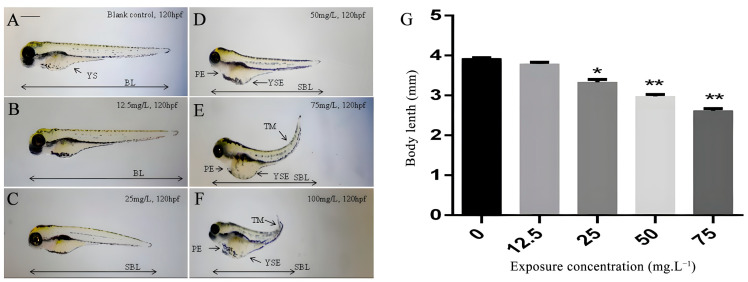
Effect of koumine on morphology and body length of zebrafish larvae (120 hpf). (**A**): Morphology of the control group (120 hpf). (**B**): Morphology of the 12.5 mg/L group (120 hpf). (**C**): Morphology of the 25 mg/L group (120 hpf). (**D**): Morphology of the 50 mg/L group (120 hpf). (**E**): Morphology of the 75 mg/L group (120 hpf). (**F**): Morphology of the 100 mg/L group (120 hpf). The koumine-exposed (0, 12.5, 25, 50, 75, and 100 mg/L) phenotype is characterized by shortened body length (SBL), yolk sac edema (YSE), pericardial edema (PE), and tail malformation (TM). (**G**): Body length of each group. At 120 hpf, koumine at 25, 50, and 75 mg/L had a significant effect on the body length of larvae (*n* = 10). Larvae in the 100 mg/L group were no longer able to meet statistical requirements due to excessive losses. Data are expressed as the means ± SD from three independent experiments compared with the control (* *p* < 0.05, ** *p* < 0.01). YS: yolk sac; BL: body length.

No significant abnormalities were observed in the 12.5 mg/L group compared to the control group (Figure 1A,B). However, in the 25 mg/L group, a significantly shorter body length (SBL) was observed, but no other morphological changes were detected (Figure 1C). Morphological deformations were observed under koumine stimulation at or above 50 mg/L, including shortening along the rostral–caudal body axis, yolk sac edemas, tail malformations, and pericardium edemas (Figure 1D–F). In addition, larvae exposed to koumine at or above 75 mg/L exhibited tail malformations, with the degree of tail curvature becoming more severe at higher concentrations (Figure 1E,F). Among these morphological alterations, the most prominent malformation was a shortened body length (Figure 1G). The larvae showed a significant inhibition of body lengths in the 25, 50, and 75 mg/L groups compared to the control group (Figure 1G).

**Figure 2:**
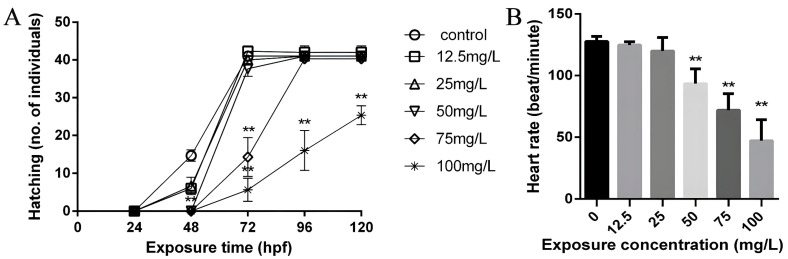
Effect of koumine on hatching in zebrafish embryo (24, 48, 72, 96, and 120 hpf) and heart rate in zebrafish larvae (72 hpf). (**A**): The number of hatchings of zebrafish embryos in the different koumine group at each time period. (**B**): At 72 hpf, koumine at 50, 75, and 100 mg/L reduced the heart rate of zebrafish larvae; data are expressed as the means ± SD from three independent experiments compared with the control (** *p* < 0.01).

Hatching is a critical period for zebrafish development, with embryos beginning to hatch after 24 hpf and completing the process by 48 hpf under normal conditions. After hatching, the heart rate can be easily measured in zebrafish by external observations of the transparent embryos, providing a reliable metric for quantifying the developmental toxicity [11,14]. In our study, we examined hatching or survival rates at five stages: 24, 48, 72, 96, and 120 hpf. Significant lethality was observed when the embryos were exposed to 100 mg/L of koumine, with only 15% of larvae surviving at 120 hpf (*p* < 0.01). However, there was no significant difference in survival between the other concentrations and the controls during embryo development (before 48 hpf), as shown in Figure 2A. The control group larvae exhibited a heart rate of approximately 130 times per minute. The heart rate of the larvae in the 12.5 and 25 mg/L koumine-treated groups remained unaffected. However, the embryos exposed to koumine at or above 50 mg/L displayed bradycardia, as shown in Figure 2B (*p* < 0.01).

### 3.2. Behavioral and Neurotoxicity of Koumine

Zebrafish embryo development occurs at a remarkable pace. Spontaneous movement is the first observable behavior, which typically starts at 17 h postfertilization (hpf). By 21 hpf, the embryos respond to touch with rapid trunk contractions. With the rapid maturation of sensory and motor systems, zebrafish larvae display highly robust and complex behaviors during the first week of development [8,17]. Therefore, locomotor activity is a commonly used quantitative endpoint to measure behavioral toxicity in zebrafish [11].

**Figure 3:**
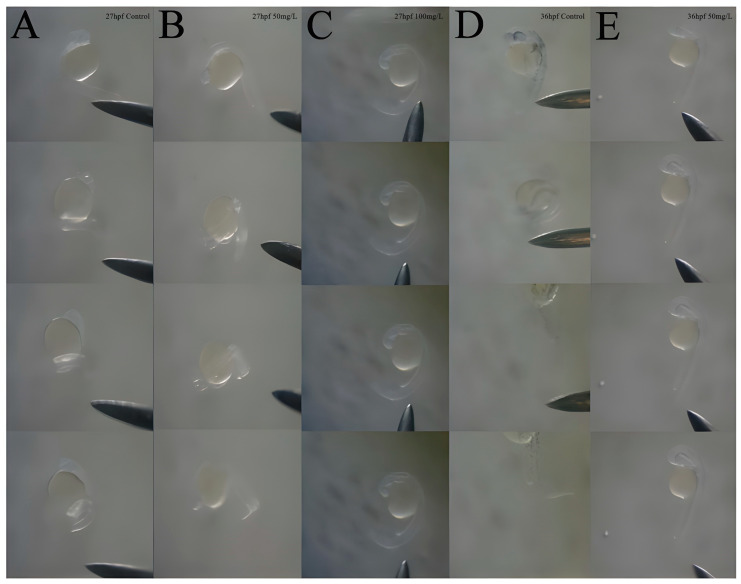
Effect of koumine on the touch response of zebrafish larvae. (**A**–**C**): The response of zebrafish larvae to touch at 27 hpf; (**D**,**E**): the response of zebrafish larvae to touch at 36 hpf.

In our study, we examined the effects of koumine exposure (at concentrations of 0, 50, and 100 mg/L) on the spontaneous behavior and escape response of zebrafish embryos by using a zebrafish behavior instrument (ViewPoint 3.5). Larval fish were detoxified at 96 h postfertilization (hpf) and cultured until 120 hpf. Subsequent observations of the total movement distance and duration were conducted. For the escape experiments, we configured the behavior instrument with the following settings: a pixel background of 24 pix, a data acquisition interval of 60 s, and a light intensity of 500 lux. We compared the movement distances across groups during the first minute of sudden illumination and the first minute following the return to darkness. Our results revealed that, at 27 hpf, larvae in the control group and the 50 mg/L koumine group exhibited normal avoidance behavior, whereas larvae in the 100 mg/L group did not respond to touch (Figure 3A–C). By 36 hpf, the larvae in the 50 mg/L group also lost their responsiveness to touch (Figure 3D,E), indicating that the behavioral toxicity of koumine to zebrafish larvae increased with prolonged exposure time and resulted in a loss of responsiveness to stimuli.

**Figure 4:**
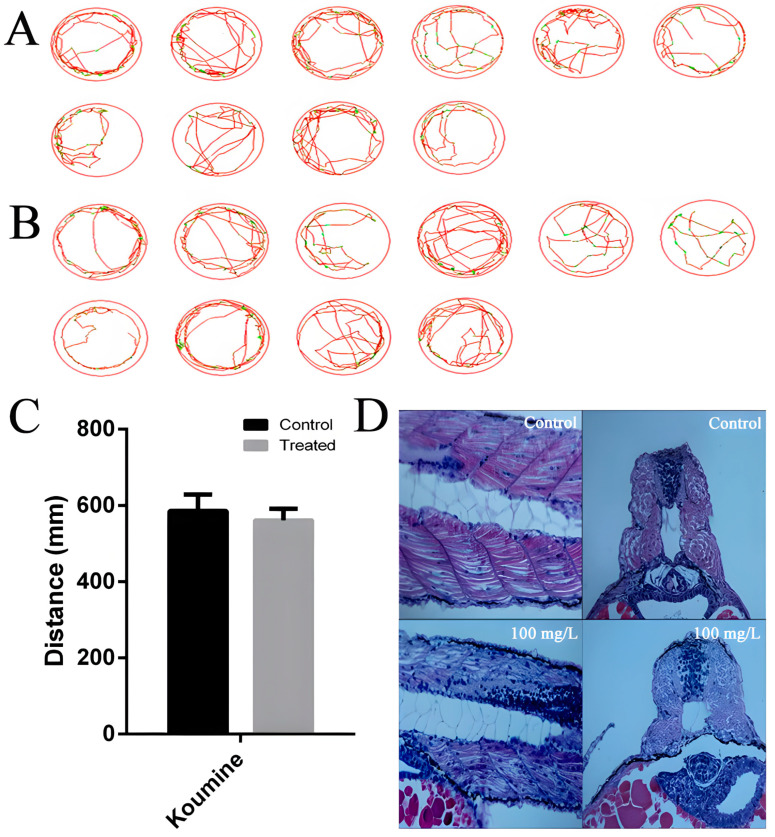
Effect of koumine on the movement trajectory, swimming distance, and muscle fibers of larvae at 120 hpf. (**A**): Movement trajectory of larvae in control group; (**B**): movement trajectory of larvae with 20 mg/L of koumine; (**C**): effect of 20 mg/L of koumine on the movement distance of larvae; (**D**): longitudinal and transverse sections of muscle tissue from larvae in 100 mg/L koumine group.

In order to further examine the effect of koumine on the motor behavior of zebrafish larvae, we examined the free-swimming behavior of zebrafish larvae by using the zebrafish behavior analyzer. At 120 h postfertilization (hpf), the free-swimming behavior of the larval fish was assessed by using a zebrafish behavior analyzer. The results indicated that 20 mg/L of koumine did not affect the locomotor activity of the larval fish; their movement patterns were indistinguishable from the control group, displaying normal thigmotaxis (Figure 4A). In the light-induced startle response experiment, koumine had no impact on either the movement distance or speed of the larval fish (Figure 4B). As depicted in Figure 4C,D, compared to the control group, the 100 mg/L koumine group exhibited disorganized and loosely arranged muscle fibers.

### 3.3. Koumine Affects ACh Content and the Activity of AChE

**Figure 5:**
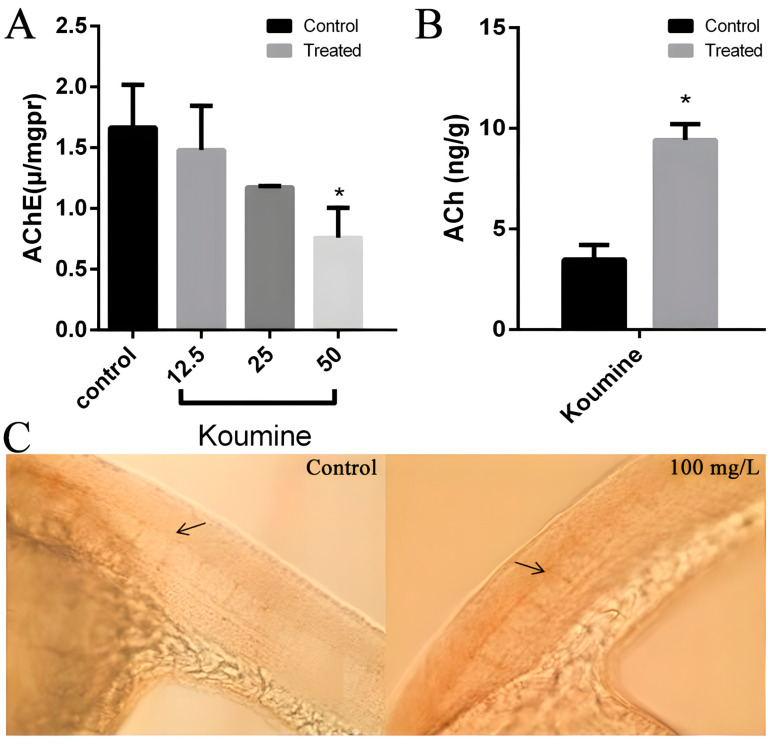
Effects of koumine on the AChE activity, ACh content, and motor neuron development of zebrafish larvae. (**A**): Effect of koumine on AChE activity in 96 hpf zebrafish larvae (* *p* < 0.05); (**B**): effect of 100 mg/L of koumine on the ACh content in 96 hpf zebrafish larvae (* *p* < 0.05); (**C**): impact of 100 mg/L of koumine on the development of primary motor neuron axons in 20 hpf zebrafish larvae.

In our experiments, koumine exhibits developmental toxicity and neurobehavioral toxicity in zebrafish larvae, so we examined AchE activity in koumine-treated zebrafish larvae as well as measured the Ach content by using LC/MS. The results showed that low concentrations of koumine (12.5, 25 mg/L) had no effect on AChE activity, while 50 mg/L of koumine had an inhibitory effect on AChE activity (Figure 5A). In addition, the ACh content of zebrafish larvae in the 50 mg/L koumine group was significantly higher compared to the control group (Figure 5B).

To further investigate the mechanism of the neurobehavioral toxicity of koumine, we stained 20 hpf embryos with the SYT2 antibody and observed the growth and development of motor neurons. The length of the motor neuron Cap axonal did not change significantly in the embryos of the koumine group (100 mg/L) compared to the control group (Figure 5C). The above results suggest that the neurotoxicity of koumine is mainly caused by influencing the ACh content and the activity of AChE without impairing motor neuron development.

## 4. Discussion

Alkaloid compounds are widely distributed, and most have significant biological activity [18,19]. For example, berberine has anti-inflammatory effects [20], reserpine can lower blood pressure, morphine has analgesic effects [21], and colchicine can treat gout [22]. It is worth noting that most of the alkaloids are accompanied by toxic effects, and the application presupposes a good toxicological test. Koumine is an alkaloid isolated from the plant *Gelsemium elegans* of the Loganiaceae, Gelsemium [19]. It is the alkaloid with the highest content but relatively low toxicity in gelsemine [23]. Experiments on various animal models have shown that koumine has good antitumor, anti-inflammatory, analgesia, and antianxiety therapeutic effects [24,25,26]. However, the toxic effects and mechanism of action of koumine are not yet clear, which limits its application.

The evaluation of toxicology can ensure the safety and effectiveness of drugs, providing basic reference materials for drug design and development [27]. In a rodent model of adjuvant-induced arthritis (AIA), both 3 mg/kg and 15 mg/kg doses of Koumine were effective in alleviating symptoms, including pain reduction, the attenuation of joint swelling, and the restoration of the M1/M2 macrophage balance within the body [19]. A 0.4 mg/kg dose of Koumine was found to be efficacious in mitigating inflammatory pain induced by formalin [26]. In an anxiety model, koumine demonstrated significant anxiolytic effects at doses of 0.5 mg/kg and 1.5 mg/kg [15]. Therapeutic efficacy against collagen-induced arthritis (CIA) in mice was observed at Koumine doses of 2, 4, and 8 mg/kg [28]. In this study, zebrafish larvae in the 50, 75, and 100 mg/L groups showed obvious malformations such as a shortened body length, pericardial edema, yolk sac edema, and curved tails. No obvious abnormalities were observed in the 12.5 and 25 mg/L groups. In addition, the hatching rate and heart rate of the high concentration groups were also significantly affected. In the neurobehavioral toxicity experiment, we selected the spontaneous coiling behavior, touch escape response, and spontaneous swimming behavior of zebrafish embryos and larvae as toxicity endpoints. The zebrafish exposed to different concentrations of koumine showed normal behavioral responses at 20 hpf. However, as the exposure time increased, the inhibitory effect of koumine on larval behavior became more apparent. The 100 mg/L group lost their touch response at 27 hpf, while the 50 mg/L group lost their touch response at 36 hpf and showed obvious convulsive behavior. By the end of the exposure, the larvae in these two exposure groups (50 and 100 mg/L) were completely paralyzed and had completely lost their ability to move. Muscle paraffin section H & E staining results showed that the muscle morphology of the larvae in the 100 mg/L koumine group were disorganized and loose. In addition, significant changes in neurotransmitter concentrations were observed. Therefore, it can be assumed that the behavioral changes in the larvae may be due to tissue damage and dysfunction of the nervous system. The larvae in the low concentration group had no obvious behavioral abnormalities, and their movement distance was not significantly different from that of the control group. Our previous research also showed that low concentrations of koumine can improve the immunity of *Cyprinus carpio* and *Megalobrama amblycephala*, while high concentrations can induce the apoptosis of *C. carpio* hepatocytes [29,30]. In contrast, studies on other model animals showed that the injection of 10 mg/kg of koumine had a better therapeutic effect. These results suggest that the positive effects of koumine are mainly presented at low concentrations, while toxic effects are seen at high concentrations. Therefore, based on our research results, the safe concentration of koumine in zebrafish embryos or larvae should be below 25 mg/L. This provides an important reference for the application of koumine.

Combined with the reported results, we found that the behavioral changes and muscle damage of embryos and larvae in the koumine exposure group were consistent with those of the *ache* zebrafish mutant [31]. Previous experiments have shown that after an injection of koumine, mice exhibit cholinergic poisoning symptoms such as reduced activity, a decreased respiratory rate, severe tremors, and clonic convulsions [32]. Studies have also shown that koumine has a significant therapeutic effect in rat models (*Rattus norvegicus*), improving learning and memory disorders and hippocampal tissue pathological changes in rats [33]. This gives us a very interesting hint that koumine may exert its neuroactivity through AChE.

Accumulated research has shown that the inhibition of AChE activity leads to the accumulation of ACh in the cholinergic synaptic cleft, causing the continuous stimulation of AChRs [34]. Damage to the cholinergic system can cause functional changes in animal tissues and organs, resulting in muscarinic (M-like), nicotinic (N-like), and central nervous system poisoning symptoms. Specific manifestations include pupil constriction, blurred vision, slowed heart rate, difficulty breathing, muscle tremors, and convulsions [35,36,37]. In this study, we measured the activity of AchE in zebrafish larvae exposed to koumine and used LC/MS technology to determine the content of the neurotransmitters in zebrafish larvae. The results showed that 50 mg/L of koumine had a significant inhibitory effect on AChE activity, and the content of ACh in larvae was significantly higher than that in the control group. These results suggest that the neurotoxic effect of koumine may be due to the inhibition of AChE activity, leading to the accumulation of ACh and resulting in developmental impairment and neurobehavioral changes in zebrafish embryos and larvae.

## 5. Conclusions

The zebrafish genome shares approximately 70% of its genes with humans, making it a widely applicable model for drug development and mechanistic studies. To date, numerous studies have confirmed the utility of zebrafish as an effective model organism for researching human-relevant drugs. In the present study, we observed the effects of koumine on development, neurobehavior, muscle tissue, and motor neurons in zebrafish. Our results showed that the toxic effects of koumine at high concentrations were caused by the inhibition of AChE activity, resulting in the accumulation of ACh, which led to changes in zebrafish embryonic and larvae development and neurobehavior. In contrast, the toxic effect of koumine at low concentrations was not obvious. The combined results suggest that the safe concentration of koumine in zebrafish larvae should be below 25 mg/L. Our study provides some basic information for the future application of koumine. However, the in-depth mechanism of action still needs to be further explored.

## Data Availability

The datasets used and/or analyzed during the current study are available from the corresponding author upon reasonable request.

## Supplementary Materials

The following supporting information can be downloaded at https://www.mdpi.com/article/10.3390/toxics11100853/s1: Figure S1: Experimental processing flow chart. Our exposure experiments started at 6 hpf, and in addition, we conducted several independent experiments. Also considering the experimental purpose and cost, we conducted separate sampling for different concentration groups at different time points during the exposure. At the later stage of exposure, the higher concentration groups such as 100 mg/L could not support all of the surviving juvenile fish due to their high toxicity. However, the combined results of our experiments still allow us to conclude that lower concentrations such as 25 mg/L, 20 mg/L and 12.5 mg/L do not produce significant toxic effects on zebrafish larvae.

## References

[1] Jin G.-L., Su Y.-P., Liu M., Xu Y., Yang J., Liao K.-J., Yu C.-X. (2014). Medicinal plants of the genus *Gelsemium* (*Gelsemiaceae*, *Gentianales*)—A review of their phytochemistry, pharmacology, toxicology and traditional use. J. Ethnopharmacol..

[2] Su Y.P., Shen J., Xu Y., Zheng M., Yu C.X. (2011). Preparative separation of alkaloids from *Gelsemium elegans* Benth. using pH-zone-refining counter-current chromatography. J. Chromatogr. A.

[3] Liu H., Ying X.U., Shi D.M., Chang-Xi Y.U. (2008). Pharmacognostical study on the *Gelsemium elegans* Benth. from Fuzhou. Strait Pharm. J..

[4] Zhang L., Lin J., Wu Z. (2003). Advances in the study on chemical constituents and pharmacology of *Gelsemium elegans* (Gardn. et Champ.) Benth. J. Chin. Med. Mater..

[5] Chen C.J., Zhong Z.F., Xin Z.M., Hong L.H., Su Y.P., Yu C.X. (2017). Koumine exhibits anxiolytic properties without inducing adverse neurological effects on functional observation battery, open-field and Vogel conflict tests in rodents. J. Nat. Med..

[6] Venard C., Boujedaini N., Mensah-Nyagan A.G., Patte-Mensah C. (2011). Comparative Analysis of Gelsemine and *Gelsemium sempervirens* Activity on Neurosteroid Allopregnanolone Formation in the Spinal Cord and Limbic System. Evid. Based Complement Altern. Med..

[7] Zhang L.-L., Wang Z.-R., Huang C.-Q., Zhang Z.-Y., Lin J.-M. (2004). Extraction and separation of koumine from *Gelsemium alkaloids*. Di Yi Jun Yi Da Xue Xue Bao.

[8] Colwill R.M., Creton R. (2011). Locomotor behaviors in zebrafish (*Danio rerio*) larvae. Behav. Process..

[9] Xu Y.-K., Liao S.-G., Na Z., Hu H.-B., Li Y., Luo H.-R. (2012). *Gelsemium alkaloids*, immunosuppressive agents from *Gelsemium elegans*. Fitoterapia.

[10] Ali S., Champagne D.L., Spaink H.P., Richardson M.K. (2011). Zebrafish embryos and larvae: A new generation of disease models and drug screens. Birth Defects Res. Part C Embryo Today Rev..

[11] McCollum C.W., Ducharme N.A., Bondesson M., Gustafsson J.-A. (2011). Developmental toxicity screening in zebrafish. Birth Defects Res. Part C Embryo Today Rev..

[12] de Esch C., Slieker R., Wolterbeek A., Woutersen R., de Groot D. (2012). Zebrafish as potential model for developmental neurotoxicity testing: A mini review. Neurotoxicol. Teratol..

[13] Bourne Y., Taylor P., Marchot P. (1995). Acetylcholinesterase inhibition by fasciculin: Crystal structure of the complex. Cell.

[14] Kimmel C.B., Ballard W.W., Kimmel S.R., Ullmann B., Schilling T.F. (1995). Stages of embryonic development of the zebrafish. Dev. Dyn..

[15] Xiong B., Zhong Z., Chen C., Huang H., Lin J., Xu Y., Yang J., Yu C. (2022). The anxiolytic effect of koumine on a predatory sound stress-induced anxiety model and its associated molecular mechanisms. Phytomedicine.

[16] Lin Y., Liu Q., Chen Z., Zheng F., Huang H., Yu C., Yang J. (2021). The immunomodulatory effect of koumine on B cells under dependent and independent responses by T cells. Eur. J. Pharmacol..

[17] Saint-Amant L., Drapeau P. (1998). Time course of the development of motor behaviors in the zebrafish embryo. J. Neurobiol..

[18] Ishikura M., Abe T., Choshi T., Hibino S. (2013). Simple indole alkaloids and those with a non-rearranged monoterpenoid unit. Nat. Prod. Rep..

[19] Kitajima M. (2022). Recent studies on chemical constituents of *Ophiorrhiza plants*. J. Nat. Med..

[20] Akbar M., Shabbir A., Rehman K., Akash M.S.H., Shah M.A. (2021). Neuroprotective potential of berberine in modulating Alzheimer’s disease via multiple signaling pathways. J. Food Biochem..

[21] van Onselen R., Downing T.G. (2021). Neonatal Reserpine Administration Produces Widespread Neuronal Losses and alpha-Synuclein Inclusions in a Rat Model. Neurotox. Res..

[22] Roth M.E., Chinn M.E., Dunn S.P., Bilchick K.C., Mazimba S. (2022). Association of colchicine use for acute gout with clinical outcomes in acute decompensated heart failure. Clin. Cardiol..

[23] Xiao S., Huang Y.-J., Sun Z.-L., Liu Z.-Y. (2017). Structural elucidation of koumine metabolites by accurate mass measurements using high-performance liquid chromatography/quadrupole-time-of-flight mass spectrometry. Rapid Commun. Mass Spectrom..

[24] Jin G.-L., Yang J., Chen W.-Q., Wang J., Qiu H.-Q., Xu Y., Yu C.-X. (2019). The analgesic effect and possible mechanisms by which koumine alters type II collagen-induced arthritis in rats. J. Nat. Med..

[25] Shoaib R.M., Zhang J.-Y., Mao X.-F., Wang Y.-X. (2019). Gelsemine and koumine, principal active ingredients of *Gelsemium*, exhibit mechanical antiallodynia via spinal glycine receptor activation-induced allopregnanolone biosynthesis. Biochem. Pharmacol..

[26] Xiong B.J., You W.B., Luo Y.F., Jin G.L., Wu M.X., Xu Y., Yang J., Huang H.H., Yu C.X. (2021). Investigation of the Possible Allostery of Koumine Extracted from *Gelsemium elegans* Benth. And Analgesic Mechanism Associated with Neurosteroids. Front. Pharmacol..

[27] Cassar S., Adatto I., Freeman J.L., Gamse J.T., Iturria I., Lawrence C., Muriana A., Peterson R.T., Van Cruchten S., Zon L.I. (2020). Use of Zebrafish in Drug Discovery Toxicology. Chem. Res. Toxicol..

[28] Li Z., Zhang J., Zhang R., Kuang Y. (2022). Extraction of koumine from *Gelsemium* Elegans Benth. and its therapeutic effect on collagen-induced arthritis in mice. Food Sci. Technol..

[29] Wang D., Wang Q., Zuo Z., Dong Z., He J., Ye X., Tang H., Zou J. (2023). Koumine induces apoptosis in Cyprinus carpio liver cells by regulating JAK-STAT and p53 signaling pathways. Fish Shellfish Immunol..

[30] Ye Q., Feng Y.Y., Wang Z.L., Zhou A.G., Xie S.L., Zhang Y., Xiang Q., Song E.F., Zou J.X. (2019). Effects of dietary Gelsemium elegans alkaloids on growth performance, immune responses and disease resistance of *Megalobrama amblycephala*. Fish Shellfish Immunol..

[31] Behra M., Cousin X., Bertrand C., Vonesch J.-L., Biellmann D., Chatonnet A., Strähle U. (2002). Acetylcholinesterase is required for neuronal and muscular development in the zebrafish embryo. Nat. Neurosci..

[32] Zhang J.-Y., Wang Y.-X. (2015). *Gelsemium analgesia* and the spinal glycine receptor/allopregnanolone pathway. Fitoterapia.

[33] Yang Z.-H., Zhang G.-M., Chen C.-Y., He J. (2021). Prenatal exposure to koumine results in cognitive deficits and increased anxiety-like behavior in mice offspring. J. Chem. Neuroanat..

[34] Murray A.P., Faraoni M.B., Castro M.J., Alza N.P., Cavallaro V. (2013). Natural AChE Inhibitors from Plants and their Contribution to Alzheimer’s Disease Therapy. Curr. Neuropharmacol..

[35] Agatonovic-Kustrin S., Kettle C., Morton D.W. (2018). A molecular approach in drug development for Alzheimer’s disease. Biomed. Pharmacother..

[36] Le L.L., Liu M.T., Song Y., Lu S.B., Hu J.N., Cao C.J., Xie B., Shi H.H., He D.F. (2018). Polystyrene (nano)microplastics cause size-dependent neurotoxicity, oxidative damage and other adverse effects *in Caenorhabditis elegans*. Environ. Sci. Nano.

[37] Nedogreeva O.A., Evtushenko N.A., Manolova A.O., Peregud D.I., Yakovlev A.A., Lazareva N.A., Gulyaeva N.V., Stepanichev M.Y. (2021). Oxidative Damage of Proteins Precedes Loss of Cholinergic Phenotype in the Septal Neurons of Olfactory Bulbectomized Mice. Curr. Alzheimer Res..

