# Supplementary figures and images for: Toxic Effects of Koumine on the Early-Life Development Stage of Zebrafish
Source: Toxics. 2023 Oct 12;11(10):853. doi: 10.3390/toxics11100853 (PMC10611223; doi:10.3390/toxics11100853)

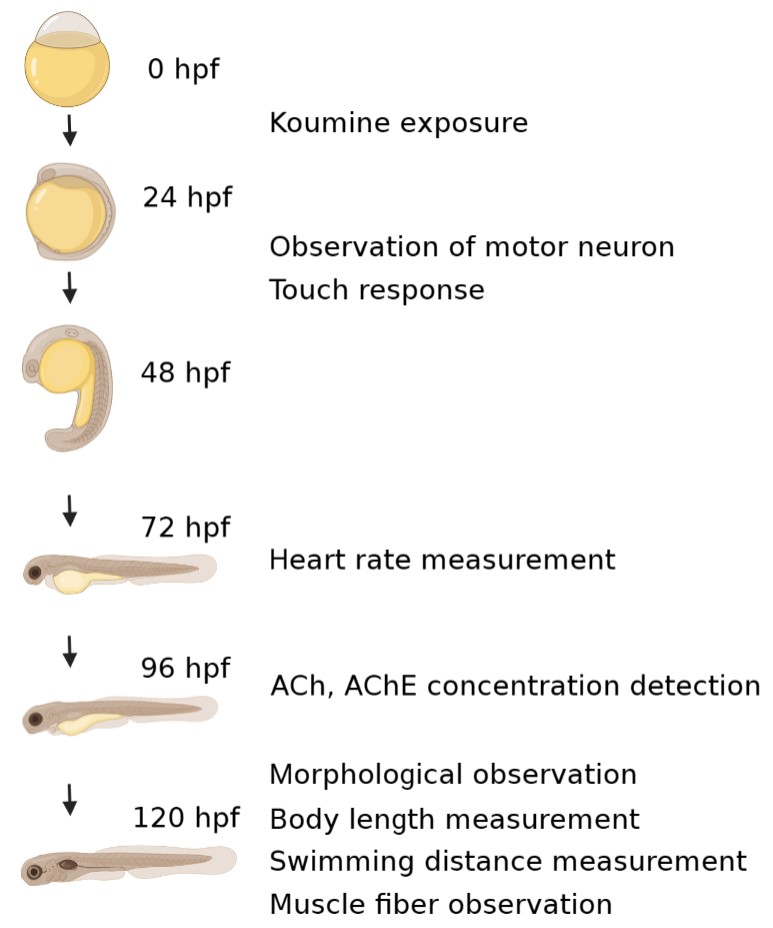

Supplement: Supplementary file 1 [file toxics-11-00853-s001.zip › toxics-2591396-supplementary.jpg]
